# Quantified Sleep: Self-Tracking Technologies and the Reshaping of 21^st^-Century Subjectivity

**DOI:** 10.12759/hsr.48.2023.21

**Published:** 2023

**Authors:** Diletta De Cristofaro, Simona Chiodo

**Keywords:** sleep, sleep crisis, quantified self, subjectivity, self-tracking technologies, sleep-tracking

## Abstract

Taking sleep-tracking as its case study, this article seeks to theorise the understandings of the self that are at stake in the the Quantified Self (QS) movement and everyday self-tracking practices by bringing together a cultural theorist’s and a philosopher’s perspectives. We situate the rise of sleep-tracking practices within the sleep crisis discourse, namely, the sense that in today’s society sleep disorders are on the rise and sleep deprivation is rife. Through analyses of self-trackers’ blogs about sleep, sleep-tracking technologies’ marketing information, and the functionalities of these devices and apps, we argue that the drive to self-improve at the heart of self- and sleep-tracking props up an understanding of the self centred around achievement. This understanding ends up devaluing sleep and risks contributing to the sleep crisis. We show how these paradoxes can be further understood from an epistemological perspective. Self- and sleep-tracking are arguably practices that seek to obtain knowledge by trading referential expert knowledge for self-referential nonexpert knowledge and that strive for self-optimisation by self-sabotaging achievement subjectivity. We conclude that the use of self-tracking technologies magnifies what is essentially a crisis of subjectivity.

## 1 Introduction^1^

A significant focus for sleep knowledge today is the so-called sleep crisis, namely, contemporary society’s presumed widespread sleep deprivation and rise in sleep disorders. According to some data, indeed, “people are getting one to two hours less [sleep] each night than their ancestors did 50–100 years ago” and “sleep pathologies are approaching epidemic levels” (Roenneberg 2013, 427). Thus, day after day, we are surrounded by news articles reminding us of the importance of sleep, warning us of the significant health risks associated with poor sleep quality and quantity, and dispensing top tips for good sleep hygiene. Sleep science, we shall see, is more cautious and divided about this supposed public health emergency than some apocalyptic headlines would suggest.^2^ Yet, hardly surprisingly for a context where we are repeatedly told that our sleep could be a matter of life and death, more and more people are self-tracking their sleep.^3^

Self-tracking encompasses practices that turn everyday experiences into data, in particular, experiences related to health and wellbeing (Neff and Nafus 2016), as in the case of sleep. Self-tracking is not a new phenomenon but, in today’s world, these practices are aided by digital technologies such as wearables and smartphones.^4^ Digital self-tracking lies at the heart of the Quantified Self (QS) movement, the phenomenon of regular self-tracking defined by *Wired* editors Gary Wolf and Kevin Kelly. The QS has a dedicated website directed by Wolf, which collects self-trackers’ projects in a section called “Show&Tell”.^5^ According to the QS movement, self-knowledge can be obtained “through numbers”,^6^ namely, through data displayed by self-tracking technologies. This idea underpins not only the QS movement’s practices but, more broadly, informal practices that characterise 21^st^- century everyday life more or less explicitly.

Taking sleep-tracking as its case study, this article seeks to theorise the understandings of the self that are at stake in the QS and everyday self-tracking practices by bringing together two different disciplinary perspectives, that of a cultural theorist and medical humanities scholar (Diletta De Cristofaro) and that of a philosopher of technology and expert in epistemology (Simona Chiodo). In the next section, “Sleep-Tracking and the Discourse of the Sleep Crisis”, De Cristofaro situates the rise of sleep-tracking within the sleep crisis discourse, framing the two as mutually reinforcing phenomena. Through analyses of self-trackers’ blogs about sleep (in particular, sleep-related “Show&Tell” posts), sleep-tracking technologies’ marketing information, and the functionalities of these devices and apps, the article’s third and fourth sections turn to explore sleep-tracking practices and their reshaping of 21^st^-century subjectivity.^7^ In the section “Sleep-Tracking: On Self-Improvement and Achievement Subjectivity”, De Cristofaro argues that the drive to self-improve at the heart of self- and sleep-tracking ultimately props up an understanding of the self centred around achievement which, in turn, ends up devaluing sleep and thus risks contributing to the sleep crisis. In the section “Sleep-Tracking: On Self-Sabotaging Self-Improvement and Achievement Subjectivity”, Chiodo shows how the paradoxes emerging from the previous section can be further understood from an epistemological perspective. In her analysis, Chiodo argues that self- and sleep-tracking are practices that seek to obtain knowledge by trading referential expert knowledge for self-referential nonexpert knowledge and that strive for selfoptimisation by somehow self-sabotaging achievement subjectivity. The conclusion summarises our argument about sleep-tracking practices and the production of 21^st^-century subjectivity, suggesting that the use of self-tracking technologies magnifies what is essentially a crisis of subjectivity.

## 2 Sleep-Tracking and the Discourse of the Sleep Crisis

“Sleep crisis”, “sleep debt”, “societal insomnia”, “sleep epidemic”—these are all synonyms for the same phenomenon, namely, the sense that contemporary society suffers from chronic sleep deprivation and an increasing number of sleep disorders. “Community studies”, a recent article in *Science* maintains, “have documented widespread sleep deprivation. Adults in the US sleep just 6.1 hours per night when objectively measured, well below the 7 to 9 hours recommended by experts. Evidence is emerging that sleep duration and quality are even lower in developing countries and among the poor in rich countries” (Rao et al. 2021, 530). This comes at a significant price for health and wellbeing, including impaired alertness and performance, which lead to more accidents, as well as long-term damages such as depression, obesity, diabetes, cancer, and heart diseases. As a RAND report puts it, “insufficient sleep increases mortality risk by up to 13%” (Hafner et al. 2017). Thus, according to some experts, sleep has now become a public health emergency (Barnes and Drake 2015; Lockley and Foster 2011).

However, not all experts agree that contemporary society is in the grip of this public health emergency. For some, like neuroscientist Jim Horne, “sleep debt is overstated, as the great majority of us have sufficient sleep, especially as our 7-hour average sleep has changed little over the last century” (2016, viii). Yet, even those who contest the sleep crisis thesis highlight how widespread this discourse is (Horne 2016, vii). As sociologist Simon J. Williams points out, there is a “readiness and willingness, within professional and popular if not lay culture, to frame or translate all manner of problems and issues into sleep-related matters; a process which itself engenders a sleep ‘crisis’ of sorts” (2005, 137).

Establishing whether or not contemporary society is in the grip of a sleep crisis goes beyond the scope of this article and of our expertise as scholars in the humanities. But, we argue, it is productive to see the rise of sleep-tracking as closely linked with the diffusion of the sleep crisis discourse. The two phenomena are mutually reinforcing, as perfectly encapsulated by the sleep-tracking app Rise, which uses sleep debt as its main metric and whose website explicitly references the sleep crisis, stating “We started Rise because we’re in the midst of an insufficient sleep epidemic”.^8^ Indeed, the widespread use of both sleep-tracking technologies and of the sleep crisis rhetoric can be understood through the intertwined frameworks of medicalisation and healthicisation.

In Peter Conrad’s classic definition, “Medicalization describes a process by which nonmedical problems become defined and treated as medical problems, usually in terms of illnesses or disorders”; whereas “with healthicization, behavioral and social definitions are advanced for previously biomedically defined events”, turning “health into the moral” (1992, 209; 223). The broad adoption of self-tracking, including sleep-tracking, is at least in part due to the process of *“biomedicalization,* which is the extension of medical or biological explanations for the way things are”. For this process “carves a groove in our collective imagination that makes close measurement of the body both conceivable and desirable” (Neff and Nafus 2016, 18-19; emphasis in original). Healthicisation is also a contributing factor in the rise of self-tracking: we are shamed for supposedly unhealthy behaviours that are seen as “br[eaking] the rules of a biomedicalized world” (Neff and Nafus 2016, 19) and nudged to self-track to correct these behaviours. The discourse of the sleep crisis participates in this shaming, for it frames individual sleep issues as a matter of public health and safety.

The discourse of the sleep crisis is similarly shaped by medicalisation and healthicisation. On the one hand, this discourse pathologises sleep, “transform[ing it] into a medical problem through the language of disorder” (Meadows et al. 2018, 176).^9^ This medicalisation occurs not just within the loci of institutional medicine but also beyond them, thanks to the prominence that the sleep crisis has in the media and contemporary culture more broadly,^10^ as well as to digital technologies, such as sleep-tracking devices and apps, which can provide useful data about our sleep and even help us identify sleep disorders, but also risk inflating the pathologisation of sleep (Meadows et al. 2018, 175-176). A case in point is so-called “orthosomnia”, “the perfectionist quest to achieve perfect sleep”, whereby self-trackers develop an unhealthy fixation on improving/perfecting their wearable sleep data,^11^ even though much more sophisticated and reliable ways of “quantifying” sleep, such as polysomnography, identify no problems with these patients’ sleep (Baron et al. 2017). On the other hand, as Williams notes, there are “increasing trends towards the healthicisation of sleep, where lifestyle choices and individual responsibility in the interests of good sleep hygiene and the pursuit of sleepsmart habits loom large” (2005, 154). In this respect, an important subtext of the discourse of the sleep crisis, which constantly reminds us of the risks of poor sleep quality and quantity, is the exhortation to make lifestyle and behavioural adjustments to ensure optimum sleep for health. The desire for optimising sleep, and thus health and wellbeing, is a crucial motivation for using sleep-tracking technologies.

The position of these technologies within the discourse of the sleep crisis is an ambiguous one, however. Sleep-tracking devices and apps feature both as a possible solution to the crisis—namely, tracking sleep allows us to identify our sleep problems and/or pushes us to make adjustments to our habits in order to ensure better sleep—and, at the same time, as a contributing factor to the crisis itself. For in addition to the already mentioned potential by-product of sleep-tracking that is orthosomnia, there is a fundamental tension between sleep and technology that lies at the core of a much-repeated origin story for the sleep crisis.

To put it with one of most prominent proponents of the sleep crisis thesis, Harvard sleep physician Charles Czeisler, “technology has effectively decoupled us from the natural 24-hour day to which our bodies evolved, driving us to go to bed later” (2013). With “technology” Czeisler is thinking specifically of light consumption but numerous other scholars point out that in the discourse of the sleep crisis it is, more broadly, technological modernity that is profoundly at odds with sleep.^12^ As Czeisler maintains, while electric light has interfered with our sleep ever since its widespread adoption in the nineteenth century, plummeting costs of electricity in the second half of the twentieth century have meant that its use has greatly increased which, in turn, has been paralleled by a significant rise in sleep deficiency. In the 21^st^ century, the situation is made even worse by the fact that LEDs, widely used in the screens of our digital devices, are rich in blue light, which is “typically more disruptive to circadian rhythms, melatonin secretion and sleep than incandescent lighting” (Czeisler 2013). It seems paradoxical, therefore, that we are trying to fix our sleep issues with digital technologies, which sleep hygiene advice repeatedly tells us to avoid at bedtime.^13^

Ultimately though, the discourse of the sleep crisis reinforces the sense of the need for sleep-tracking by virtue of its being about health and safety risks. As sociologist Deborah Lupton argues, discourses of risk within public health contribute to the production of a specific type of subjectivity. The self is understood as calculating and self-regulating, and as acting in accordance with imperatives of public health and risk-avoidance that are self-imposed, rather than externally imposed, as they have been internalised in what Lupton identifies as neoliberal governmentality. As she writes, “risk may be understood as a governmental strategy of regulatory power by which populations and individuals are monitored and managed through the goals of neo-liberalism [sic]” (1999, 88). The reference to neoliberalism is important, for in this socio-political and economic system, with the progressive erosion of the welfare state, health becomes more and more a matter of individual responsibility (Osborne 1997). Individuals are thus driven by a “quest for self-improvement” (Lupton 1995, 9), while their risk-avoiding strategies are coded as a “moral enterprise relating to issues of self-control [and] self-knowledge” (Lupton 1999, 92-93). Health, as already discussed through healthicisation, turns into a moral imperative and individuals not engaging in self-improvement and risk-avoidance are framed as irrational, immoral, and even failures. That is why many people enthusiastically embrace self-tracking technologies, which are tools supporting the self-knowledge, self-regulation, and self-improvement at the core of their understanding of the self.

## 3 Sleep-Tracking: On Self-Improvement and Achievement Subjectivity

“It’s human to want to get better at what we do, not just at work but in life”, confesses a developer introducing a blog post listing the many apps and tools he uses in his “journey of self-tracking and continuous improvement”, including a wearable band tracking sleep patterns (Molinari). In line with the type of subjectivity Lupton identifies, one driven by a quest for self-improvement, there is a direct connection between the developer’s everyday self-tracking practices and improvement, for “that which is measured improves” (Molinari). Thus, his purpose for sleep-tracking is to “analyze [his] data monthly and decide if it’s good enough or if [he] should change [his] routines to improve the time and/or quality of [his] sleep” (Molinari).^14^ If the motto of the QS is “self-knowledge through numbers”, this self-knowledge is rarely an end in and of itself but, rather, is leveraged to improve something, be it health or other aspects of life.

While Molinari’s sleep-tracking seems to be mostly about improving his health, as this practice is included in his post’s “Body and Health” category, it is telling that he is a self-proclaimed “productivity aficionado” and that his post features in the blog section of a productivity app. The link between sleep, sleep-tracking, and productivity is an important one, and crucial to our theorising of the understandings of the self at the core of the QS and everyday self-tracking practices. This link is embedded in the very discourse of the sleep crisis, where sleep deficiency is often framed as a threat to the economy. To put it with RAND reports, “sleep matters” because “insufficient sleep can result in large economic costs in terms of lost GDP and lower labour productivity” (Hafner et al. 2016).^15^ The same link is also central to how sleep-tracking apps and devices are marketed.

Take the example of the Ōura smart ring, which, with its companion app, promises to be a “sleep lab wrapped around your finger” by providing “24/7 heart rate monitoring” and by analysing “deep sleep, REM sleep, light sleep, nightly heart rate, bedtime schedule, and more”.^16^ Ōura taps into the QS’s understanding of data as tools for self-improvement for, as the company puts it in a tweet, “You can’t improve what you can’t measure” (@ouraring, August 5, 2021). On the surface, Ōura seems to encourage sleep-tracking, and thus sleep-improvement, in the name of a “sleep-positive agenda” (Williams 2011, xiv), namely, it appears to want to counteract the sleep crisis by championing sleep and rest as key to a healthy life. As the smart ring’s website explains, “Ōura can detect when you’re tired, unwell or under stress by reading the changes in your key body signals associated with stress. It then automatically adjusts your daily goals to *put your rest and recovery first”* (emphasis mine) through in-app exhortations like “don’t push it” or “try taking it easy today”. Indeed, “by looking at your heart rate and skin temperature”, Ōura tells you “if you’re taking enough breaks throughout the day to *get the recovery your body needs*” (emphasis mine).

However, rather than valuing sleep on its own terms, Ōura ultimately upholds an instrumental view of rest as a tool for maximising productivity, performance, and achievement during the day. As the company’s Facebook bio puts it, “Improve sleep. Perform better”.^17^ This view is similarly upheld by other sleep-tracking devices, such as the WHOOP wearable, whose tagline is “Performance Optimization”.^18^ In turn, the instrumental understanding of sleep and rest at the core of these technologies risks contributing to the sleep crisis^19^—be this an actual decline in sleep quality and quantity, or simply a perception of sleep being under threat in today’s society—by perpetuating a harmful “sleep-negative agenda” that devalues sleep and privileges waking life (Williams 2011, xiii). This agenda is perfectly summed up by the Rise app, which claims that the “real sleep goal [is] being at your best during the day” (notice, too, the app’s name).^20^

While Ōura is mainly marketed as a sleep-tracker, the smart ring and companion app center around “3 daily scores to guide you”: the sleep score, which answers the question “how’d you sleep last night?”, the activity score, which answers the question “how are you balancing your activity, inactivity, and rest?”, and the readiness score, which answers the question “how much can you and your body take on?”. With taglines like “Harness your potential with clear and actionable insights” and “Start your day smarter”, it is apparent that the sleep score—and the goal of “perfecting] your sleep” associated with this score—is subordinated to the readiness score, namely, being ready to face the day productively and perform better (and the fact that the readiness score also features in other popular sleep-trackers, from Fitbit to AutoSleep, only emphasises how influential the instrumental view of sleep as a tool for maximising performance is). Indeed, the nap detection function, which automatically records one’s naps, is explained on Ōura’s website by showing how adding naps to the sleep score fundamentally improves the readiness score. As Ōura’s Twitter posts indicate, sleep is thus framed as a way to “set ourselves up for success” (@ouraring, March 4, 2002), since “You snooze [,] you win” (@ouraring, December 9, 2021). Ultimately, Ōura’s core philosophy and marketing strategy, reflected in the broader landscape of sleep-tracking technologies, is that “A good night’s sleep can transform your day. Sleep more. Achieve more” (@ouraring, January 7, 2022). This slogan perfectly encapsulates how the self-improvement facilitated by self-tracking’s “self-knowledge through numbers” props up an understanding of the self revolving around achievement, in other words, an “achievement subjectivity” (Han 2015).

As philosopher Byung-Chul Han explains, “Twenty-first century society is no longer a disciplinary society, but rather an achievement society” whose inhabitants are “entrepreneurs of themselves”. In a disciplinary society, subjects are driven by externally imposed imperatives, or, by “the negativity of compulsion [of] *Should”* (Han 2015, 8). Achievement society, instead, “discard[s] negativity”: “Unlimited *Can* is the positive modal verb of achievement society”, so that the “Prohibitions, commandments, and the law [of disciplinary society] are replaced by [self-imposed] projects, initiatives, and motivation” (Han 2015, 8-9).^21^ This shift from disciplinary society to achievement society is due to the “drive to maximize production”, since “Beyond a certain point of productivity, disciplinary technology—or, alternatively, the negative scheme of prohibition—hits a limit. To heighten productivity, the paradigm of disciplination is replaced by the paradigm of achievement, or, in other words, by the positive scheme of *Can*” (Han 2015, 9). This is why we can detect such an emphasis on productivity and performance even in sleep-tracking technologies, which, if anything, should foster rest and inactivity. Driven by the seemingly endless potentials of “the positive scheme of *Can*”, achievement-subjects see themselves as “performance-machine[s]” in constant competition with each other and, indeed, with oneself (Han 2015, 30). To put it with a testimonial for the now-defunct wearable Jawbone, which used to track movement and rest: “Competing with myself really inspires me” (Kleinman 2017).

The pervasive language of goals and achievements in self-tracking, together with the continuous availability of data that allow to measure one’s performance, suggest that these technologies can be interpreted as the tools through which achievement-subjects work on improving themselves as their own entrepreneurial projects. Given their dedication to self-track, and thus improve, “every facet of life … 24/7/365” (Wolf 2009), members of the QS can arguably be understood as achievement-subjects *par excellence*. In this context, sleep becomes just another achievement to “unlock”, to draw on the vocabulary of gamification that often characterises self-tracking technologies.^22^ And, since “gamification strips away everything that makes a game *playful* … retaining just the repetitive punishment-reward dynamics—the grinding, the ‘treadmilling’, the unimaginative *labor* involved in leveling up” (Berson 2015, 130; first emphasis in original, second mine), sleep, too, effectively risks becoming akin to labour (something that sleep-tracking technologies already gesture towards through their emphasis on performance), one more thing to tick off from the achievement-subject’s endless to-do list.

This constant compulsion to achieve, improve, and compete with oneself and others is, indeed, not devoid of problems. The “achievement-subject grinds itself down”, Han writes, “It is tired, exhausted by itself, and at war by itself” (2015, 42), an aspect that a “Show&Tell” post on the QS website poignantly captures. Explaining why she stopped self-tracking “after 40 measurements a day for 1.5 years”, Alexandra Carmichael avows that her “self-worth was [too] tied to the data”, to achieving “the right numbers”, so much so that self-tracking had become “an instrument of self-torture” (2010). Achievement subjectivity, which, we have argued, is what is at stake in the self-tracker’s “self-knowledge through numbers”, can become utterly unbearable, as further explored in the next section. It is for this reason that ours is a “burnout society”, to put it with the title of Han’s book, and that there is a widespread sense of a sleep crisis.

## 4 Sleep-Tracking: On Self-Sabotaging Self-Improvement and Achievement Subjectivity

The paradoxes that have emerged, from trying to improve sleep by using self-tracking technologies that can worsen it to trying to value sleep by actually devaluing it as something functional to achievement subjectivity, seem to arise from a more radical paradox that, from a philosophical perspective, may be read, first, as an epistemological issue and, second, and again, as an issue of subjectivity. In what follows, we shall especially address the testimonies on sleep published in the “Show&Tell” section of the QS website, in that they are the clearest representatives of a general attitude characterising not only regular self-tracking but also less regular practices in our everyday life.

From an epistemological perspective, the paradox may be described as follows:^23^ on the one hand, self-improvement and achievement subjectivity should be guaranteed by “numbers”, namely, by data displayed by self-tracking technologies. Yet, on the other hand, “numbers” do not mean anything without expert knowledge, especially when self-trackers are not doctors (as it is mostly the case), but nevertheless try to self-diagnose without any medical knowledge or experience and, therefore, without the resulting ability to read one’s particular numbers within the context of one’s general health. More precisely, nonexpert self-trackers seem to fall into two kinds of epistemological errors. First, they seem to trade (referential) expert knowledge for (self-referential) nonexpert knowledge, as we have already begun to see in the previous section with orthosomnia. For instance, “my doctor had done what he could and medicine had done what they could and I had to do something for myself. […] so I’ve kind of cured myself of Crohn’s disease”.^24^ Even more radically, “I started going to my doctor, but I wasn’t really believing in that method, because I didn’t see a really problem-solving method. […] my doctors. They can’t help me, so I’ll try and find these methods of my own health condition”.^25^ The move from expert knowledge (which is referential as it results from the scientific community) to nonexpert knowledge (which is self-referential as it does not result from the scientific community) arises from a kind of reductionism according to which “numbers” are *per se* expert knowledge without requiring not only the context but also, and especially, the capacity to understand it (which means, for instance, that “data driven health solution[s] using numbers”, namely, “living by numbers”,^26^ may even lead to dangerous misunderstandings of one’s health condition if no context and no capacity to understand it are at play).

And dangerous misunderstandings also seem to emerge through the paradoxical gap that characterises the relationship between what one feels about oneself and what one knows, namely, believes to know, about oneself “through numbers”. Even though, on the one hand, self-trackers can feel they sleep perfectly well (“I thought I was a really good sleeper”), on the other hand, if, according to numbers, “it turned out I wasn’t”,^27^ then numbers win over one’s feelings, namely, one’s subjectivity is shaped more by numbers (which are nonexpertly understood) than by one’s feelings. As a self-tracker puts it, “I think that data is going to be the way that we understand who we are”, and, while “how I feel about the quality of that sleep isn’t always correlated with or supported by the data measured by my Basis watch”, “when I see the numbers from Basis contradict my positive assessment of my rest, my sense of having rested well is suddenly undermined as if those Basis numbers somehow have the power to make me doubt my own experience”^28^ (from a phenomenological perspective, we may say that the objective body, even though nonexpertly understood, wins over the subjective body).

Finally, the vicious circle between one’s numbers and oneself seems to stress a kind of individualism that affects not only one’s epistemological dimension (namely, one’s self-referential nonexpert knowledge, as we have seen) but also one’s ethical dimension (namely, a kind of solipsism in which one’s existential counterpart is no one but their self-tracking technologies). Thus, individualism shows up both epistemologically (“the most important takeaway for me in my tracking experience is that general assumptions don’t work for individuals”,^29^ which is surprisingly opposite to what the glorious history of the scientific method can show) and ethically (when “personal science” ends up making one think that “I’m not that interested in coming up with general solutions for other people, I want to improve my condition”^30^).

The second kind of epistemological error is that self-trackers seem to overvalue correlations. Paradoxically enough, even though they want nothing but certainty, they seem to naively (again, nonexpertly) think not only of numbers as certainty, as we have seen, but also of correlations as a kind of certainty, namely, as causations. More precisely, the QS movement’s motto “self-knowledge through numbers”, specifically numbers that “are making their way into the smallest crevices of our lives” (Wolf 2009, no page number) as “the facts” we “can live by” (Wolf 2010, no page number), actually means correlations that, as such, cannot underpin certainty at all. While, again, the glorious history of the scientific method can show that correlations do not mean causations, namely, a kind of certainty, self-trackers seem to be frequently obsessed with making correlations coincide with science, specifically certainty resulting from it (for instance, but examples are several, “I was curious to see what science would tell me. So, I used SPSS to find correlations between my 35 nights”^31^), even though at times they realise that correlations may mean nothing meaningful (“Finally we get the correlation and that is the one interesting thing with correlations, that if you keep looking long enough you will find one”^32^) and at other times they realise that correlations may mean something obvious, which does not require the effort of years of self-tracking (“I found there is a surprisingly large correlation between quality of sleep and how I was feeling that day, which shouldn’t be that surprising”^33^). Thus, from our perspective, what is most interesting to understand is the possible answer to the following question: why do self-trackers seem to self-sabotage, somehow, the epistemological underpinning of their effort of self-optimisation? More precisely, why do they seem to paradoxically strive, on the one hand, to self-optimise as achievement-subjects and, on the other hand (and at the same time), to somehow self-sabotage as achievement-subjects?

Trying to answer this question makes us move from the epistemological issue to the issue of subjectivity, which may be captured by the following question: what may self-tracking technologies reveal about the reshaping of their users’ subjectivity, from regular self-tracking to less regular practices in everyday life? In what follows, in order to try and answer these questions, we shall address two kinds of issues, which may also be read as consequences of the epistemological errors we have identified in previous pages.

The first issue to address has to do with the kind of obviousness that, as we have seen, frequently results from self-tracking. Interestingly enough, obviousness seems to be considered more as something reassuring, a confirmation, rather than as something critical from an epistemological perspective. As for obviousness in general, we can read, among others, testimonies such as “what have I learned? […] resting heart rate [is influenced by] illness like I showed, but also things like stress”;^34^ “You can see that sleep effects [sic] your day, the day effects [sic] your sleep”;^35^ “if it is colder in my bedroom up to a certain point I will be able to sleep better”;^36^ “the less sleep that I am having actually affects the stress level as well”;^37^ “[in terms of] positive impacts [on sleep], for me that was eating light, eating regular, being active in a healthy way, working out, fun with friends”;^38^ “I also found that food effects [sic] your clarity levels”^39^ and “So still a kind of an obvious finding”.^40^ As for obviousness as reassuring, that is, obviousness as a confirmation, we can read in particular, among others, testimonies such as “my sleep is very fragmented. The Zeo confirmed it and I already guessed that”;^41^ “[I] asked my computer […] what makes me happy and he says well you have to be healthy, just be active, have a good night sleep and don’t be stressed. And that’s pretty nice to know that actually what you suppose […] will make you happy does make you happy”;^42^ “what I found is quite what I expected […] and for me the most important finding is [that] the quality of your day actually starts the day before because when you have a good night’s sleep then you can have a good day”;^43^ “my second conclusion […] might seem obvious to you but it’s interesting that it’s validated from the data”^44^ and “it does help if I get fit and I sleep better, and I found that to be true”.^45^ Thus, why is obtaining obviousness worth self-tracking for years and years? The answer that seems to emerge from self-trackers’ testimonies is precisely that obviousness is reassuring and confirming. More precisely, obviousness is reassuring and confirming to the highest degree precisely if it results from an epistemological attitude that, by virtue of being self-referential, as we have seen, is exposed to potentially disruptive disconfirmations to the lowest degree.

Thus, our working hypothesis may be the following: the more we use self-tracking technologies as self-referential self-confirmation, the more the way we use them seems symptomatic of a kind of crisis we experience as achievement-subjects, a crisis we have already begun to explore in the previous section. More precisely, we seem to try to unburden ourselves of the increasingly unbearable burden of being achievement-subjects.

If our working hypothesis makes sense, then it may serve as a possible way to read the series of paradoxes we have described (trying to improve sleep by using self-tracking technologies that can worsen it, trying to value sleep by actually devaluing it as something functional to achievement subjectivity, trying to obtain knowledge by trading referential expert knowledge for self-referential nonexpert knowledge, and trying to strive for self-optimisation by somehow self-sabotaging achievement subjectivity). The series of paradoxes described seems to exemplify that we somehow try to self-sabotage ourselves as achievement-subjects. And the reason for our self-sabotage may be that achievement subjectivity more and more becomes the increasingly unbearable burden we wish to shed. The tool of our self-sabotage may be technology, specifically the kinds of technologies that we can use not only solipsistically (namely, without disconfirmations, as we have seen) but also, and especially, as what can unburden us by somehow automating our decisions and actions, which can more and more move from resulting from us as (autonomous) achievement-subjects (to be finally judged as such) to resulting from (automated) technologies as avatars of our achievement subjectivity (not to be finally judged as such).^46^

Thus, may we read the reassuring obviousness resulting from our self-tracking technologies as a kind of equally reassuring automation that can more and more unburden us of the burdens of our autonomous decisions and actions?

Actually, the second issue to address, namely, the issue of subjectivity, seems to give us reasons to develop our working hypothesis further. Among self-trackers’ testimonies, we find the following: “So you pledge money towards your goals, we collect your data, send reminders and make a graph and if you don’t do what you said you were going to do we take your money”.^47^ It is no coincidence that the testimony’s title is “Extreme productivity”, for productivity, as discussed in the previous section of this article, is somewhat synonymous with achievement subjectivity. The testimony quoted exemplifies the QS movement’s typical attitude: “if you want to set a goal you should measure it otherwise you’re not going to make it”.^48^ And the reason why “you’re not going to make it” is that individuals seem to move from being thought of as driven by (autonomous) reasons, as Kant would put it, to being thought of as driven by (automated) numbers, from scores to money, as Pavlov might put it. Paradoxically enough, achievement-subjects somehow continue to be achievement-subjects not by bearing autonomy’s burden (for instance, by deciding and acting for ethical reasons, among what Kant [1785 and 1788] would define as reasons), but by trading it for a kind of classical conditioning produced by automation. And, again, classical conditioning essentially sabotages achievement subjectivity, in that achieving goals driven by scores and money (which is something that even Pavlov’s dog can do) is not the same as achieving goals driven by autonomous reasons, from epistemological reasons to ethical reasons and sense-making reasons (which is something that individuals, and not dogs, can and should be capable of doing).

## 5 Conclusion

Focussing on sleep-tracking as a case study, we have considered how self-tracking technologies, together with several other digital technologies that similarly make our decisions and actions less autonomous and more automated, are reshaping our sense of self. This is not a novel phenomenon in itself, if we consider the variety of ways in which humans have used technology for millennia to reshape themselves, but, in the case of self-tracking and other digital technologies, the novelty has to do with both a special intensity and a special focus on trading human autonomous decisions and actions for technological automation. 21^st^-century subjectivity has thus emerged as the site of contradictory impulses and paradoxes, which self-tracking technologies both channel and enhance. The self-improvement facilitated by the self-tracker’s “self-knowledge through numbers” props up a subjectivity driven by imperatives of endless achievement that can become increasingly unbearable and may even lead to burnout. These pressures on the contemporary self can be further understood both in epistemological terms (as a crisis caused by the overwhelming burden of being individuals able to pursue perfect knowledge) and in ethical terms (as a crisis caused by the overwhelming burden of being individuals able to pursue perfect morality). Ultimately, self-tracking technologies bring a magnifying glass to the crisis of the contemporary self, which the discourse of the sleep crisis, be this actual or just perceived, also serves to vocalise.

## Footnotes

1 The article’s introduction (section 1) and conclusion (section 5) are co-authored. The article’s second and third sections are written by Diletta De Cristofaro; the article’s fourth section is written by Simona Chiodo. This approach brings into dialogue two different disciplinary perspectives: that of a cultural theorist and medical humanities scholar who is working on a project on representations of sleep and the sleep crisis in contemporary culture (De Cristofaro; see https://www.writingsleep.com/), and that of a philosopher who is studying the epistemological and ethical implications of emerging technologies (Chiodo). According to their respective specialisms, De Cristofaro advances the article’s argument through close readings, the approach at the core of literary and cultural studies, Chiodo develops instead a philosophically-informed methodology based around questions.

2 Cf., for instance, this headline from the British tabloid newspaper *The Sun:* “NIGHTMARE: Getting less than seven hours’ sleep a night could KILL you as experts warn Brits are in the grip of a ‘sleep crisis’“ (Larbi 2019).

3 “It is estimated that 10% of US adults use a wearable fitness/sleep tracking device on a regular basis, and 50% would consider purchasing one” (Baron et al. 2017, 351).

4 In this article, “self-tracking” and “sleep-tracking” refer to contemporary practices aided by digital technologies.

5 See https://quantifiedself.com/ (accessed March 21, 2022). See also Wolf 2010.

6 See https://quantifiedself.com/ (accessed March 21, 2022).

7 By using the word “reshaping”, we do not mean to subscribe to a deterministic view whereby technology causes societal outcomes. Rather, technology and society evolve together, each shaping the other.

8 See https://www.risescience.com/ (accessed March 15, 2022). Cf. also AutoSleep, which similarly tracks sleep debt; https://autosleepapp.tantsissa.com/clock/wellness (accessed March 15, 2022).

9 Not all aspects of sleep and sleep disorders are medicalised in the same way of course. For nuanced analyses of the medicalization of sleeplessness as insomnia see, for instance, Coveney et al. 2018; Kroker 2022. What we are interested in tracing in this article is, however, the broader sense of sleep as a problem signalled by the discourse of the sleep crisis. We are also interested in the relationship between the medicalisation of sleep and the socio-economic context in which this takes place. Barbee et al.’s suggestion that neoliberalism’s productivity imperative “foster[s] an environment wherein medicalization not only survives but thrives” (2018) chimes with the argument we develop in the following pages.

10 For an analysis of the sleep crisis in contemporary literary culture, see De Cristofaro forthcoming.

11 Note orthosomnia’s emphasis on self/sleep-improvement and achieving sleep goals, which the next section expands upon.

12 Cf. for instance Scrivner 2014.

13 Cf. for instance: “patients who are overly reliant on their sleep tracking devices may also have other problematic technology use patterns, such as smartphone notifications or calls interrupting sleep and working or watching TV from a laptop or phone at night” (Baron et al. 2017, 354).

14 What makes the sleep data “good enough” for the self-tracker is an important question, which we turn to explore, together with the epistemological paradoxes this question uncovers, in the next section of this article.

15 See also, for instance, Hillman et al. 2018.

16 See https://ouraring.com/; unless otherwise stated, all quotations referring to Ōura in what follows are taken from this website (accessed March 15, 2022).

17 See https://www.facebook.com/ouraring/ (accessed March 16, 2022). This instrumental view of sleep is not just typical of sleep-tracking technologies but characterises, more broadly, digital technologies that foster sleep. Take, for instance, the app Pzizz (https://pzizz.com/), which uses “dreamscapes” to help people sleep better at night and take power naps during the day. Pzizz’s tagline is, tellingly, “Sleep, Nap, Focus”, which again encapsulates how sleep is understood not as valuable in and of itself but only as a tool to maximise alertness and productivity.

18 See https://apps.apple.com/us/app/whoop-performance-optimization/id933944389 (accessed March 16, 2022).

19 As argued elsewhere, “Since our work patterns are often to blame for poor sleep quality and quantity, a constant emphasis on productivity and performance doesn’t exactly seem to make for a good solution to our supposed sleep crisis” (De Cristofaro 2022).

20 See https://www.risescience.com/blog/sleep-score (accessed 17 March 2022).

21 Similarly to Lupton, for Han, the context of the production of this entrepreneurial self, with its internalised imperatives and demands, is neoliberalism, which can also be understood as the breeding ground for the rise of self-tracking practices. As Williams, Coveney, and Meadows argue, “these digital technologies mesh or chime with existing processes of individualisation and responsibilisation, and neoliberal values of enterprise and enhancement” (2015, 1048).

22 On the potential health issues that gamifying sleep may bring, see Meadows et al. 2018, 176.

23 As extensively argued in Chiodo 2022 and forthcoming.

24 See https://quantifiedself.com/show-and-tell/?project=750 (accessed on 21 March 2022).

25 See https://quantifiedself.com/show-and-tell/?project=563 (accessed on 21 March 2022).

26 See https://quantifiedself.com/show-and-tell/?project=218 (accessed on 21 March 2022).

27 See https://quantifiedself.com/show-and-tell/?project=574 (accessed on 23 March 2022).

28 See https://quantifiedself.com/show-and-tell/?project=756 (accessed on 23 March 2022).

29 See https://quantifiedself.com/show-and-tell/?project=15 (accessed on 23 March 2022).

30 See https://quantifiedself.com/show-and-tell/?project=181 (accessed on 23 March 2022).

31 See https://quantifiedself.com/show-and-tell/?project=1086 (accessed on 23 March 2022).

32 See https://quantifiedself.com/show-and-tell/?project=704 (accessed on 23 March 2022).

33 See https://quantifiedself.com/show-and-tell/?project=750 (accessed on 23 March 2022).

34 See https://quantifiedself.com/show-and-tell/?project=1082 (accessed on 23 March 2022).

35 See https://quantifiedself.com/show-and-tell/?project=847 (accessed on 23 March 2022).

36 See https://quantifiedself.com/show-and-tell/?project=704 (accessed on 23 March 2022).

37 See https://quantifiedself.com/show-and-tell/?project=324 (accessed on 23 March 2022).

38 See https://quantifiedself.com/show-and-tell/?project=110 (accessed on 23 March 2022).

39 See https://quantifiedself.com/show-and-tell/?project=549 (accessed on 23 March 2022).

40 See https://quantifiedself.com/show-and-tell/?project=916 (accessed on 23 March 2022).

41 See https://quantifiedself.com/show-and-tell/?project=51 (accessed on 23 March 2022).

42 See https://quantifiedself.com/show-and-tell/?project=847 (accessed on 23 March 2022).

43 See https://quantifiedself.com/show-and-tell/?project=784 (accessed on 23 March 2022).

44 See https://quantifiedself.com/show-and-tell/?project=15 (accessed on 23 March 2022).

45 See https://quantifiedself.com/show-and-tell/?project=349 (accessed on 23 March 2022).

46 As extensively argued in Chiodo forthcoming.

47 See https://quantifiedself.com/show-and-tell/?project=803 (accessed on 24 March 2022).

48 See https://quantifiedself.com/show-and-tell/?project=549 (accessed on 24 March 2022).
